# Supplementary material for: Unpacking Psychological Vulnerabilities in Deaths of Despair
Source: Int J Environ Res Public Health. 2023 Jul 31;20(15):6480. doi: 10.3390/ijerph20156480 (PMC10418686; doi:10.3390/ijerph20156480)
Supplement: Supplementary file 1 [file ijerph-20-06480-s001.zip › ijerph-2399228-supplementary.pdf]

Supplementary Table S1.

Multinomial Logistic Regression Predicting Death of Despair and Death of Cancer by the levels of Purpose in Life, Education, and Gender (Reference = Death of Heart Disease)

	**Death of Despair**		**Death of Cancer**	
	OR [95% CI]	*p*	OR [95% CI]	*p*
Age	0.932 [0.910-0.953]	0.000	0.963 [0.943-0.982]	0.000
Women (vs. men)	1.003 [0.646-1.558]	0.988	1.404 [0.962-2.049]	0.079
Non-Hispanic white (vs. other race/ethnicity)	1.529 [0.874-2.677]	0.137	0.966 [0.598-1.561]	0.886
College degree (vs. high school or less)	1.905 [0.851-4.265]	0.117	1.114 [0.556-2.233]	0.761
Household income, logged	0.989 [0.873-1.120]	0.860	1.036 [0.926-1.159]	0.538
Married (vs. unmarried)	0.740 [0.465-1.175]	0.202	1.423 [0.946-2.141]	0.090
Working (vs. not working)	1.086 [0.672-1.755]	0.736	1.324 [0.875-2.005]	0.184
Purpose in life: lowest tertile	1.031 [0.561-1.898]	0.921	1.063 [0.614-1.840]	0.828
Purpose in life: middle tertile	0.792 [0.415-1.513]	0.480	1.043 [0.592-1.835]	0.885
Purpose in life: Highest tertile (reference)	---	---	---	---
College degree × Purpose in life–lowest tertile	5.267 [1.491-18.611]	0.010	1.372 [0.495-3.797]	0.543
College degree × Purpose in life–middle tertile	2.207 [0.596-8.168]	0.236	1.116 [0.428-2.915]	0.822
2 Log Likelihood = 1452.69, Nagelkerke R^2 = 0.145				

Note: Cause of death (death of despair, death of cancer and death of heart disease) were categorized based on multiple cause of deaths and underlying cause of death information from NDI Plus (2004-2022). Death of despair included death by suicide, alcoholism, or drug addiction. OR = Odds ratio, CI = Confidence interval.

Supplementary Table S2.

Multinomial Logistic Regression Predicting Death of Despair and Death of Cancer by the levels of Positive Relations with Others, Education, and Gender (Reference = Death of Heart Disease)

	**Death of Despair**		**Death of Cancer**	
	OR [95% CI]	*p*	OR [95% CI]	*p*
Age	0.928 [0.907-0.950]	0.000	0.960 [0.941-0.980]	0.000
Women (vs. men)	0.923 [0.590-1.444]	0.726	1.329 [0.903-1.955]	0.149
Non-Hispanic white (vs. other race/ethnicity)	1.557 [0.890-2.722]	0.121	0.943 [0.583-1.524]	0.810
College degree (vs. high school or less)	0.665 [0.285-1.553]	0.346	0.963 [0.481-1.927]	0.916
Household income, logged	0.972 [0.858-1.102]	0.661	1.028 [0.918-1.151]	0.627
Married (vs. unmarried)	0.668 [0.418-1.066]	0.091	1.346 [0.891-2.034]	0.158
Working (vs. not working)	1.029 [0.640-1.654]	0.907	1.259 [0.834-1.903]	0.273
Positive relations: lowest tertile	0.601 [0.318-1.133]	0.116	0.599 [0.341-1.053]	0.075
Positive relations: middle tertile	0.822 [0.449-1.507]	0.527	0.974 [0.576-1.648]	0.922
Positive relations: Highest tertile (reference)	---	---	---	---
College degree × Positive relations−lowest tertile	3.412 [1.023-11.377]	0.046	2.374 [0.837-6.734]	0.104
College degree × Positive relations−middle tertile	0.649 [0.191-2.202]	0.488	1.053 [0.414-2.674]	0.914
2 Log Likelihood = 1360.14, Nagelkerke R^2 = 0.145				

Note: Cause of death (death of despair, death of cancer and death of heart disease) were categorized based on multiple cause of deaths and underlying cause of death information from NDI Plus (2004-2022). Death of despair included death by suicide, alcoholism, or drug addiction. OR = Odds ratio, CI = Confidence interval.

Supplementary Table S3.

Multinomial Logistic Regression Predicting Death of Despair and Death of Cancer by the levels of Personal Growth, Education, and Gender (Reference = Death of Heart Disease)

	Death of Despair		Death of Cancer	
	OR [95% CI]	*p*	OR [95% CI]	*p*
Age	0.931 [0.909-0.952]	0.000	0.962 [0.943-0.982]	0.000
Women (vs. men)	0.961 [0.617-1.498]	0.862	1.358 [0.927-1.990]	0.117
Non-Hispanic white (vs. other race/ethnicity)	1.600 [0.916-2.795]	0.099	0.978 [0.605-1.581]	0.928
College degree (vs. high school or less)	0.439 [0.190-1.018]	0.055	1.044 [0.534-2.041]	0.900
Household income, logged	0.976 [0.862-1.104]	0.699	1.033 [0.923-1.156]	0.575
Married (vs. unmarried)	0.736 [0.463-1.169]	0.194	1.386 [0.920-2.088]	0.119
Working (vs. not working)	1.018 [0.633-1.638]	0.940	1.276 [0.845-1.927]	0.247
Personal growth: lowest tertile	0.659 [0.364-1.194]	0.169	0.843 [0.491-1.448]	0.537
Personal growth: middle tertile	0.675 [0.354-1.287]	0.233	0.879 [0.493-1.566]	0.662
Personal growth: Highest tertile (reference)	---	---	---	---
College degree × Personal growth−lowest tertile	3.411 [1.028-11.320]	0.045	1.161 [0.412-3.271]	0.778
College degree × Personal growth−middle tertile	2.267 [0.677-7.585]	0.184	1.373 [0.526-3.583]	0.517
2 Log Likelihood = 1367.96, Nagelkerke R^2 = 0.135				

Note: Cause of death (death of despair, death of cancer and death of heart disease) were categorized based on multiple cause of deaths and underlying cause of death information from NDI Plus (2004-2022). Death of despair included death by suicide, alcoholism, or drug addiction. OR = Odds ratio, CI = Confidence interval.

Supplementary Table S4.

Multinomial Logistic Regression Predicting Death of Despair and Death of Cancer by the levels of Positive Affect, Education, and Gender (Reference = Death of Heart Disease)

	**Death of Despair**		**Death of Cancer**	
	OR [95% CI]	*p*	OR [95% CI]	*p*
Age	0.930 [0.908-0.952]	0.000	0.964 [0.944-0.984]	0.001
Women (vs. men)	0.982 [0.633-1.525]	0.937	1.413 [0.966-2.066]	0.074
Non-Hispanic white (vs. other race/ethnicity)	1.408 [0.807-2.458]	0.229	0.937 [0.578-1.520]	0.792
College degree (vs. high school or less)	0.584 [0.261-1.307]	0.190	1.346 [0.747-2.425]	0.322
Household income, logged	0.986 [0.870-1.117]	0.819	1.032 [0.922-1.156]	0.584
Married (vs. unmarried)	0.764 [0.482-1.210]	0.251	1.466 [0.975-2.203]	0.066
Working (vs. not working)	1.096 [0.681-1.764]	0.705	1.438 [0.950-2.178]	0.086
Positive affect: lowest tertile	0.746 [0.409-1.359]	0.338	1.016 [0.608-1.700]	0.951
Positive affect: middle tertile	1.366 [0.776-2.405]	0.280	1.149 [0.685-1.927]	0.599
Positive affect: Highest tertile (reference)	---	---	---	---
College degree × Positive affect: lowest tertile	3.637 [1.013-13.052]	.048	1.375 [0.485-3.899]	0.549
College degree × Positive affect: middle tertile	1.187 [0.380-3.707]	.768	0.681 [0.268-1.731]	0.420
2 Log Likelihood = 1354.05, Nagelkerke R^2 = 0.143				

Note: Cause of death (death of despair, death of cancer and death of heart disease) were categorized based on multiple cause of deaths and underlying cause of death information from NDI Plus (2004-2022). Death of despair included death by suicide, alcoholism, or drug addiction. OR = Odds ratio, CI = Confidence interval.
